# Transforming Education Using Virtual Reality: Geriatrics Clerkship Before and During Pandemic

**DOI:** 10.1093/geroni/igab046.2216

**Published:** 2021-12-17

**Authors:** Pamela Saunders

**Affiliations:** Georgetown University, WASHINGTON, District of Columbia, United States

## Abstract

Since 2006, the Georgetown University School of Medicine has offered a two-week elective in Geriatrics for third-year medical students. Students rotate through diverse clinical experiences, including general geriatrics, geriatric neurology, physical medicine & rehabilitation, memory disorders, Parkinson’s and dementia, and palliative care. In addition, students learn about arts, humanities & ethics, communication skills, and taking the patient’s perspective. In Fall 2019, pre-pandemic, we added virtual reality (VR) experiences focused on hearing & vision loss, Alzheimer’s disease, and end-of-life conversations created by Embodied Labs. Curricular goals included increasing students’ empathy and sensitivity, decreasing ageism & stereotyping, and increasing clinical knowledge. Findings suggest regardless of pandemic (pre vs. during) or modality (in-person vs. Zoom) that after participating in the VR labs, students are slightly more comfortable taking care of older adult patients with dementia as well as hearing & vision loss, and participating in end-of-life conversations.

